# Impact of the primary care residents on the productivity of the ambulatory health centres in Portugal: a cross-sectional study

**DOI:** 10.1186/s12909-022-03528-y

**Published:** 2022-06-16

**Authors:** Ivo Reis, Gonçalo Envia, Paulo Santos

**Affiliations:** 1 UCSP Cantanhede, ACeS Baixo Mondego, ARS Centro, Coimbra, Portugal; 2 ACeS Sintra, ARS Lisboa E Vale Do Tejo, Queluz, Portugal; 3 Department of Medicine of Community, Information and Health Decision Sciences, Faculty of Medicine, CINTESIS - Center for Health Technology and Services Research, University of Porto, Porto, Portugal

**Keywords:** Internship and Residency, Health Care Evaluation Mechanisms, Primary Health Care, Family practice

## Abstract

**Background:**

The presence of residents in Primary Care health centres may influence their operational results.

**Aim:**

To examine the relationship between the presence of residents and the results of the evaluation in Portuguese Primary Care Health Centres.

**Methods:**

We conduct a cross-sectional study, comparing the results achieved by the mainland Portuguese Primary Care Health Centres measured by the Global Performance Index (*Índice de desempenho global* – IDG) by the presence of General & Family Medicine residents in training. Analysis took into consideration the distribution by region and typology of the health centres.

**Results:**

We evaluated 906 units, 55.7% involved in the training of General & Family Medicine residence. The presence of residents was associated with higher Global Performance Index values (77.3 vs 57.6; *p* < 0.001). The higher difference was found in the less developed Personalized Health Care Units and in the region of Lisbon and Tagus Valley.

**Conclusion:**

The presence of residents in training is a contributing factor in the productivity of the Primary Health Care facilities. It may model the asymmetry in the performance of Portuguese Health Centres.

## Background

In Portugal, the Ministry of Health provides postgraduate medical training in the regimen of medical residence, in collaboration with the National Medical Association (Decree-law no. 13/2018, published in 26/02/2018). During the four years postgraduate training programme, the trainees will progress in knowledge, skills and attitudes, to reach a level of proficiency compatible with the autonomous exercise of the specialty. In this course, the resident is tutored by the training supervisor, who is responsible for monitoring, guiding, evaluating and propose corrections to the formative pathway to achieve a high medical performance according the specialist profile. This relationship constitutes a very intimate direct dyad during the residence.

The residence of General and Family Medicine has a total duration of 4 years (Ministerial ordinance no. 125/2019, of Ministry of Health, published in 30/04/2019). It presents 3 different internships and several specific training both practical and theoretical. The first year is dedicated to the fundamentals and pillars of the specialty. The second and third years comprise the scope, diversity and complexity of General and Family Medicine. It includes the training in essential aspects of hospital specialties like internal medicine, paediatrics, gynaecology, obstetrics, psychiatry, orthopaedics and trauma, surgery and several optative internships according to the specific needs of each resident. The last year is dedicated to the integration of skills, management of practice and clinical governance in Primary Health Care (PHC), covering and integrating the knowledge, attitudes and skills of previous training and adding the dimensions of clinical and health management and governance. During all time, residents are active members of the health team with impact on its functioning and its results.

The Portuguese PHC system is based on a network of health centres distributed nationwide since 1970s. From the initial pyramidal structure, where the decision level was mainly top-down, PHC organization evolved to a decentralized frame, closer to the providers and real needs of the population. These third generation Health Centres were created in 1999 by Decree-law no. 157/99, and aimed to modernize and adapt the response of PHC. The operationalization of the principle created the Family Health Units (*Unidade de Saúde Familiar* – USF) with the publication of Decree-Law no. 298/2007 [1–3].

The new USFs were established under the principle of organizational, functional and technical autonomy, and responded to the individual medical assistance needs of a population between 4 and 20 thousand people. Together with public health teams, community care and paramedical assistance, USF integrated the 55 Portugal mainland Health Centre Groups, enclosing about 200,000 individuals each. USFs are mainly an organizational development from the classical health centres based on the providers’ individual work to the implementation of real teamwork in which medical staff, nurses and secretaries responsibly assume their role in health team, with solidarity, cooperation, conciliation and participatory management, through well-established pathways. The objective is to continuously improve quality, which also implies a culture of regular and objective evaluation and re-evaluation, and the expansion of the model to the entire national territory [4].

The USF presents in three distinct types (Table 1). The USF-A model corresponds to teams still in the maturing phase, allowing the improvement of teamwork and the development of the practice of internal evaluation, using a matrix of organizational indicators from structure and process dimensions. The USF-B model units present greater organizational maturity and an effective practice of team working, with more rigorous performance requirements under independent accreditation processes. These units have access to a pay for performance system, including financial incentives attributed to the institution and individual prizes, which may ascend to about two thirds of the base salary. The USF-C model units are potentially supplemental to the national health service units, open to the social, cooperative and private sectors. These have never been implemented in practice. The Personalized Health Care Units (*Unidade de Cuidados de Saúde Personalizados* – UCSP) are those health centres that did not transfer to the USF model, representing about a quarter of all PHC units.

**Table 1:** Typology of Primary Care Health Centres in Portugal

	UCSP	USF-A	USF-B
Legislation	Decree-Law n.º 28/2008	Decree-Law n.º 73/2017	Decree-Law n.º 73/2017
Team constitution	Does not apply	Doctors, nurses and administrative staff	Doctors, nurses and administrative staff
Recruitment	Public tender	Spontaneous and voluntary	Spontaneous and voluntary
Standard Evaluation	Does not apply	Minimal Requirements (DiOr)	Strict Requirements (DiOr)
Team size	Does not apply	Adjusted to assistance coverage	Adjusted to assistance coverage
Team cooperation	Does not apply	Yes	Yes
Technical Autonomy	No	Yes	Yes
Organizational autonomy	No	Yes	Yes
Payment	Monthly Salary	Monthly Salary	Pay for Performance over monthly salary
Funding	Public	Public	Public
Patients per doctor (2019)	1487 ± 413	1655 ± 166	1795 ± 116

*UCSP* Personalized Health Care Units, *USF-A* Type A Family Health Units, *USF-B* Type B Family Health Units, *DiOr* Grid for the diagnosis of organizational development in Primary Health Care (Diagnóstico de Desenvolvimento Organizacional nos Cuidados de Saúde Primários)

Regardless of the typology of the functional units, the 2007 reform consolidated the assessment of performance as the key for developing the PHC. The daily assistance started to integrate the quality measured by a set of indicators related to the structure, the processes and the results, in a perspective of continuous improvement, with potential gains in the health results [5]. At the same time, the pay for performance was introduced in the USF-B, focusing the attention in the quantitative aspects of the measures rather than in the underlying objectives, losing the perspective of quality improvement to the evaluation grid [6]. As Turcotte-Tremblay stated, [7] this system presents the risk of manipulation of medical registers, such that the reported quantity and quality of care differ from what is actually delivered to correspond to the contract, and consequently causing dissatisfaction and demotivation of the providers. To break it, the index of global performance (Índice de Desempenho Global – IDG) was settled, closer to the complexity of the patients’ approach in PHC. IDG is based on the evaluation of five areas of production in PHC: (1) performance in the care provided, weighing 50% for the total index, (2) available services – 10%, (3) organizational quality – 20%, (4) professional education and training – 10%, and (5) scientific production – 10%. More than the univocal analysis of the different indicators, this IDG integrates the measures of performance in a multidimensional structure closer to the real practice and defocused from the specific indicators [8, 9]. The IDG allows a quick evaluation of the overall production of the Health Unit, its operation and the attained results, in addition to being an effective tool for comparison between health units.

Although the sharing of the medical knowledge with other doctors and with our students is an ethical commitment, multiple constraints are barriers to teaching, both from the organization related issues, like inadequate facilities or little available time, as from the lack of professional skills for education or even personal motivation to do it. Some facilities do not provide any training at all. The impact of the presence of residents in the health services production has been addressed mostly from the cost evaluation, showing a large benefit at the hospitals and marginal or even non-existent in the PHC [10]. It is relevant to characterize this relationship from the point of view of health gains and service productivity. Our aim is to establish the impact of the presence of residents in the productivity of PHC health teams, measured by the IDG index and considering the different typologies of organizational models and the geographical distribution.

## Methods

We conducted a cross-sectional study, including all the Portuguese mainland health centres, in the year of 2019, before the perturbation introduced by COVID-19 pandemic.

We studied several publicly available variables, including number of residents and specialists by unit, geographic distribution (according Nomenclature of Territorial Units for Statistics—NUTS II), typology of the health centre (UCSP, USF-A and USF-B), and the global performance index (IDG). All data referred to the end of 2019, representing the final evaluation of the whole year, as published on the official web page of the Portuguese Ministry of Health at BI-CSP (ID of Primary Health Care) and collected in early 2021.

As pointed above, the IDG corresponds to the weighted sum of five sectorial indices of the areas of performance of clinical practice, services provided, organizational quality, professional training and scientific activity. Annually, the Ministry of Health, through the Central Administration of the Health System (ACSS, IP) defines which indicators are included in the construction of each sectoral index, considering the National Health Plan, the Regional Plans and the Local Health Plans, as well as the rules and other guidelines issued by the Directorate-General for Health. It varies between 0 and 1, being translated as a percentage, with higher values reflecting better overall performance of the health units.

This study was exempt from previous appraisal of Ethics Committee since it was based on a document analysis, using publicly available secondary data, without direct or indirect intervention on individuals. Nevertheless, the ethical commitment is rather important than the law that regulates it [11]. Data were analysed anonymously and treated with respect for the ethical principles of the Declaration of Helsinki and the Oviedo Convention.

Statistical analysis included frequency and association measures. It was performed in SPSS Statistics 24.0 ®.

We checked the normality of continuous variables by Kolmogorov–Smirnov test. The inferential analysis used chi-square test, Mann–Whitney U and Pearson’s correlation. The multivariate analysis used a logistic regression model to compare the first to the forth quartile of the IDG distribution. We accepted an alpha error of 0.05.

## Results

We analysed data from 906 health centres (40.0% UCSP, 31.8% USF-A and 30.2% USF-B), 55.7% involved in the general and family medicine residence programme, with a mean of 3 residents ([1-16], interquartile range [IQR] = 3) per unit. Most of UCSP did not have any residents (73.5%). The great number of residents were allocated to the USF-B (4.8 per unit), which represented 49.7% of the units involved in the residence training (Table 2).

**Table 2:** The distribution of the General and Family Medicine residents, by the typology of the health centres

Typology	Units without residents	Units with residents	Total of residents	Residents/unit
UCSP	253	91	247	2,7
USF A	125	163	480	2,9
USF B	23	251	1203	4,8

*UCSP* Personalized Health Care Units, *USF-A* Model A Family Health Units, *USF-B* Model B Family Health Units

The median of IDG was 70.7 in mainland Portugal ([17.5–94.3], IQR = 29.3). The health centres involved in the residence programme presented higher values of IDG than those who had no residents (77.3; IQR = 18.7 vs 57.6; IQR = 28,4; *p* < 0,001). The presence of residents in the unit was associated with higher performance of UCSP and USF-A but not in USF-B health centres (Table 3).

**Table 3:** Distribution of global performance index (IDG) values by the organizational typology and the presence of residents

	IDG Median (IQR)	Units with residents Median (IQR)	Units without residents Median (IQR)	*P*
UCSP	51.7 (23.6)	57.6 (23.1)	48.7 (21.1)	0.002
USF-A	71.5 (20.9)	73.4 (19.9)	68.4 (23.0)	0.013
USF-B	83.0 (8.0)	83.0 (8.5)	83.1 (6.9)	0.454

*IQR* Interquartile Range, *UCSP* Personalized Health Care Units, *USF-A* Model A Family Health Units, *USF-B* Model B Family Health Units, *IDG* Global Performance Index

Analysing by regions, we realize that there were significant differences in the UCSP and USF-A of the Lisbon and Tagus valley region and in the USF-A of the North region, where the presence of residents was associated with higher IDG (Table 4).

**Table 4:** Distribution of global performance index (IDG) values by geography (NUTS II) and the presence of residents

	IDG Median (IQR)	Units with residents Median (IQR)	Units without residents Median (IQR)	p
**UCSP**				
North	59.2 (24.5)	65.4 (19.5)	57.7 (27.2)	0.08
Centre	50.2 (18.2)	54.1 (25.9)	49.0 (16.4)	0.647
Lisbon and Tagus valley	43.4 (16.6)	55.0 (22.3)	42.5 (14.8)	0.045
Alentejo	58.6 (25.8)	64.8 (19.6)	55.6 (26.3)	0.460
Algarve	41.4 (20.1)	49.6 (20.5)	38.0 (18.6)	0.130
**USF A Model**				
North	78.5 (13.9)	80.8 (10.2)	75.1 (15.3)	0.015
Centre	67.2 (19.9)	68.2 (18.7)	66.2 (20.1)	0.788
Lisbon and Tagus valley	64.5 (23.0)	70.4 (20.8)	60.9 (22.5)	0.021
Alentejo	53.8 (20.9)	70.3 (23.2)	50.0 (33.9)	0.073
Algarve	66.6 (21.8)	66.6 (24.0)	66.8 (40.7)	0.699
**USF B Model**				
North	84.6 (6.8)	84.6 (6.9)	84.7 (24.5)	0.960
Centre	83.0 (7.8)	83.0 (8.3)	- *	0.563
Lisbon and Tagus valley	81.3 (8.9)	81.2 (9.7)	82.0 (6.9)	0.358
Alentejo	77.0 (6.4)	77.0 (6.4))	- **	-
Algarve	82.0 (21.2)	82.0 (21.2)	- **	-

^*^ *n* = 1; ** *n* = 0; *UCSP* Personalized Health Care Units, *USF-A* Model A Family Health Units, *USF-B* Model B Family Health Units, *IDG* Global Performance Index

Also, the higher number of residents was correlated with higher IDG (*ρ* = 0.275; *p* < 0,001), especially when we look at the number of residents by specialist (*ρ* = 0.279; *p* < 0,001).

In a multivariate analysis comparing the first quartile of the IDG distribution with the forth quartile, the presence of residents in the PHC health centres was associated to higher values of IDG (OR = 3.580; 95%CI: 1.294–9.906; *p* = 0.014), regardless the number of residents per health centre (*p* = 0.319), the number of residents per specialist (*p* = 0.092) and the number of patients per doctor (*p* = 0.225). Other variables with significance were the typology (*p* < 0.001), the geographic location (*p* < 0.001) and the proportion of patients without an assigned family doctor (*p* = 0.010).

## Discussion

The presence of residents in Portuguese primary care health centres is associated with greater productivity as measured by the Global Performance Index (IDG), a composite index of production measurement in PHC used in Portugal that encompasses the dimensions of performance of clinical practice, services provided, organizational quality, professional training and scientific activity. This relationship is verified regardless of the typology (although not significant in the USF-B model units) and the region, although both contribute actively for the differences.

Searching the literature, there are only few studies about the impact of residents and residence on the productivity of PHC facilities. An open study of Bridges et al., in 1999, showed an increase in costs due to the integration of interns in the surgical work related to an increase in operative time caused by the lower dexterity of residents [12]. In 2014, Hiller did not find significant differences in financial costs using a paired methodology [13]. Sibia (2020) showed an increase in the surgical time, but a compensatory decrease in the inpatient time, without significant overall difference in the global costs, and, most important, in the clinical outcomes [14]. Lewis, in his PhD thesis of 2021, identify the collaboration in the training of junior clinicians as a factor associated with best performing PHC [15]. The meta-analysis of Bourne reviewed 17 articles and showed students may have a neutral or positive effect on allied health patient activity levels and clinical time [16].

In this study, the PHC facilities involved on the residency programs showed significant higher productivity levels. The relation is strong, although the cross-sectional design does not allow to establish any causality between both. Several possible factors may explain our findings.

In Portugal, the regional directions of residences are responsible for the allocation of vacancies to residents, defining a national yearly map according to the medical residency regulation. All units are eligible for being training centres if they fulfil the required conditions of structure, processes and results as settled by the Portuguese Medical Association. Vacancies are filled by public tender on a national basis. With rare exceptions, there are no constraints in the country that health teams cannot overcome to apply to receive trainees.

Following the 2007 PHC reform, many health centres evolved to higher organization patterns with the creation of Family Health Units (USF), applying for USF-A recognition and then for USF-B, as they achieved the quality standards for qualification. The type B USFs present greater maturity in both processes and teamwork. Consequently, they are expected to be better prepared to receive residents, both in physical resources, as in the organization and human resources. The higher standards and the best conditions draw the attention of the best candidates looking for the top settings to take their PHC residences, favouring positive discrimination, in addition to being potentially more motivated, factors known to have a direct impact on the performance [11]. Nevertheless, residents are integrated into regular work contributing little to its growth. The USF-A are less mature and UCSP even less. The presence of residents may boost the reorganization of all staff and resources to accomplish the required standards, thus impacting the results. Nevertheless, the USF-A has also a strong motivation for changing in the legitimate expectation of progression to the B model, where the salary supplement based on both the production and productivity complements the precarious base salary. In addition, it is possible that residents are a real reinforcement of the workforce in needier units, where there are many patients without an assigned family doctor, as in several UCSP, but not in USF, especially in the regions of Lisbon and Tagus Valley and Algarve, which have significantly lower coverage rates than the rest of the country (75.6% and 82.6%, respectively, whereas the national rate is 87.5%; *p* < 0.001).

In the Portuguese context, the presence of residents in PHC health centres is associated with better performance in the UCSP and USF-A. It seemed to contribute to the development and maturity of the less developed health teams, without conditioning the performance of USF-B. This effect is rather evident in the larger regions of the North and Lisbon and Tagus Valley, which present more patients and more facilities.

We did not aim to study other potentially confounding variables, such as the structure of the health team, its differentiation, its stability, total time of teamwork and geodemographic issues because they were not available at the time. Meanwhile, the covid-19 pandemic brought to light some of these constraints and allowed us to quantify them in new indicators for each health unit. Portuguese PHC, like in almost all countries, suffered a huge impact on the regular effectiveness and we must wait for some regularization of the normality to review its functioning.

On the other hand, the cross-sectional nature of this study does not allow to assign the causality relationship. As we already stated, we cannot assume that the presence of residents improves the production, nor we cannot indicate that residents search for the best units to realize their internships. We believe that there may exist a crossing of both dimensions. The health centre organization creates conditions for the residence to be a daily challenge of intellectual stimulation, permanent updating, innovation and development, able to leverage the team itself and actively promote high-quality standards within it.

In this sense, the presence of residents in all health centres should be encouraged, as a mean to balance the quality. Of course, we know that although medical education and training are an ethical and deontological duty of all, [17] not everyone is available to do it or even prepared for it [18]. However, even there will always be one main host institution, it is possible that residents circulate by other health centres in the same region or not, with gains for their own training and for the health outcomes in general. This point was already introduced in the last review of PHC residence in Portugal two years ago and we look forward to evaluating it at the end of the current residence.

These results help to characterize the role of residents in the functioning of health centres. We all understand that medical education and training are a crucial investment for health services. They are one of the fundamental pillars of the structure of health services, allowing to leverage quality and consequently to improve health in our patients in the medium and long term. Our results show that this effect can also be verified in the short term in a training system based on clinical practice, as it is in Portugal. On the other hand, they rise the concern about the potential dependence of the services on the presence of residents. The needs for PHC supply must be provisioned by stable and committed staff, and not depend on the presence or effective work of trainees. They must always be supernumerary. We found a great heterogeneity due to organization (types of USF), to the lack of resources (patients without assigned family doctors) and even to the geographical dispersion through the country. These factors are relevant for the management decision of the health units and ultimately for the decision of national health policies. Fifteen years after the beginning of Primary Health Care Reform there are still so many inequities in the Portuguese health system, with impact on universal health coverage, one of the health-related Sustainable Development Goals settled by World Health Organization, [19] and on people’s lives.

In conclusion, our results show some asymmetry in the performance of the health centres across Portugal according to the typology and in the different regions. The collaboration in the residence of PHC may actually contribute to model this heterogeneity and to improve quality in health care. More than bringing closed answers, this study raises questions about the role of the residents, not from the administration perspective, but from the achieved health outcomes, contributing to the equity in health, to the continuous quality improvement of the team and to higher performance of the health centres.

## Data Availability

The dataset supporting the conclusions of this article is available in the private repository, in https://docs.google.com/spreadsheets/d/1NlKZBh_g7TPmwfdDn6e4544LdDGhmf9JjqCrmeY2uo4/edit#gid=0.
